# When Games Influence Words: Gaming Addiction among College Students Increases Verbal Aggression through Risk-Biased Drifting in Decision-Making

**DOI:** 10.3390/bs14080699

**Published:** 2024-08-11

**Authors:** Huina Teng, Lixin Zhu, Xuanyu Zhang, Boyu Qiu

**Affiliations:** 1 School of Health Management, Guangzhou Medical University, Guangzhou 510180, China; 2 School of Mental Health, Guangzhou Medical University, Guangzhou 510180, China

**Keywords:** gaming addition, inhibitory control, risk preference, aggression, hierarchical drift-diffusion model

## Abstract

Increased aggression due to gaming addiction is a widespread and highly publicized problem. The underlying processes by which verbal aggression, a more harmful and persistent subcategory of aggression, is affected by gaming addiction may differ from other types of aggression. In this study, data came from 252 randomly recruited current university students (50.79% male, mean age 19.60 years, SD: 1.44 years, range 17 to 29 years). Participants reported gaming addiction and different types of aggression through questionnaires. In addition, two important explanatory processes, inhibitory control, and risk preference, were measured through behavioral experiments. A Bayesian hierarchical drift-diffusion model was employed to interpret the data from the risk preference task. In contrast to previous work, the study found that inhibitory control did not significantly correlate with either gaming addiction or any form of aggression However, the drift rate, a measure of decision-making inclination under risk, partially mediates the relationship between gaming addiction and verbal aggression (but not other forms of aggression). The findings illuminate risk preference under adverse conditions as a key predictor of verbal aggression, offering avenues for early intervention and suggesting game design modifications to mitigate verbal aggression by adjusting reward mechanisms.

## 1. Introduction

Video games have become one of the most widely used forms of entertainment, but they also pose a variety of adverse effects. Approximately 300 million individuals globally engage in video gaming [1], with around 510,000 dedicated gamers in the U.S. who habitually play video games as their primary source of entertainment [2]. Video games can induce adverse effects affecting players’ cognitive [3], behavioral [4], and emotional well-being [5], but they can also yield certain positive effects on gamers [6,7]. How to utilize video games, the “double-edged sword”, while avoiding these unwanted consequences is of great importance.

In the research on the adverse consequences of video games, a range of theories and research evidence have supported that gaming addiction leads to aggression. The General Aggression Model [8] and the General Learning Model [9] have proposed that violent content in video games affects aggressive cognition [10,11] and negative emotions [12,13], leading to aggressive behaviors [14,15]. For specific evidence, Li et al. [16] identified a correlation between a higher level of Internet gaming addiction and increased aggression in a meta-analysis. In a laboratory study, Zhang et al. [17] found that exposure to violent video games was associated with more aggressive cognition and behaviors. Similar evidence can be found in a series of studies [18,19,20]. It can be argued that the relationship between gaming addiction and increased aggression has a solid theoretical and research foundation.

### 1.1. Game Addiction and Game Violence

It should be noted that the current study focused on gaming addiction but not gaming violence. Gaming addiction is an uncontrollable, excessive, and compulsive video gaming behavior that leads to impaired social or emotional functioning [21]. Game violence focuses on the impact of violent elements in-game content on players [22], whereas game addiction focuses more on the extent of players’ overdependence on the game and the impact of the game on their daily lives [23]. Mohammad et al. [23] suggested that many factors contribute to game addiction and that violent gaming is only one of them. Király et al. [24] convened 4887 gamers to validate the Ten-Item Internet Gaming Disorder Test, emphasizing the characteristics of Internet gaming disorder as a behavioral addiction that differs from a single violent gaming behavior. Therefore, the choice to study gaming addiction is focused on a specific psychological and behavioral problem, different from studying gaming violence.

While it is true that most video games contain violent content, many potential mechanisms of influence may cause game addicts to exhibit aggression in addition to the violent content in the game [25]. Studies have shown that competitive pressures [26], reward systems [27], social interactions [28], and emotional regulation [29] may contribute to players’ post-game aggression to varying degrees. Chen et al. [30] conducted three types of studies combining cross-sectional, experimental, and longitudinal approaches, and found that exposure to competitive video games increased adolescents’ aggression and impulsivity. Of these, impulsivity mediated the relevance and long-term effects of competitive video game exposure on aggression. However, there are fewer studies on inhibitory control and risk preference mediating game addiction and aggression. Therefore, the present study aimed to examine the relationship between gaming addiction and verbal aggression, with inhibitory control and risk preference examined as mediating variables.

### 1.2. Verbal Aggression

Most studies tend to generalize aggression as an overall indicator [31] and lack a detailed examination of the different dimensions of aggression, especially verbal aggression [32]. Instead, this study chose to analyze verbal aggression in depth. Compared to other types of aggression (e.g., physical aggression, anger, hostility, and self-aggression), verbal aggression is easily learned and has a more lasting effect. On the one hand, verbal aggression has a low cost and low threshold for implementation, making it easier to learn in the media [33] and transfer to real life. On the other hand, verbal aggression is more difficult to detect than physical aggression and often more persistent and profound [34], it can further induce other types of aggression [35] and lead to more harmful and lasting consequences [36].

Also, gaming addiction may increase the incidence of verbal aggression in several ways [37]. The competitive and stressful nature of video games can lead to players experiencing higher emotional activation and stress during gameplay [38]. This can diminish the player’s real-life social skills and emotional regulation, thus increasing the likelihood of verbal aggression. Therefore, it is of great importance to examine the relationship between verbal aggression and gaming addiction, as well as the role of two explanatory mechanisms (inhibitory control and risk preference) in the paths.

The long-term social and psychological impact of gaming addiction on individuals is of great importance. McNamee et al. [39] used data from a large longitudinal study of adolescents in the United Kingdom to investigate the relationship between social media use and emotional and behavioral outcomes. The study found that prolonged social media use (more than 4 h per day) was significantly associated with poor emotional well-being and increased behavioral difficulties, in particular a reduced sense of self-worth and an increased incidence of hyperactivity, inattention, and behavioral problems. In a four-wave longitudinal study, Parrish et al. [40] found that television time predicted inhibitory control and academic change in early childhood. Over the long term, individuals with gaming addiction may fall into isolation and rejection, increasing the risk of social adjustment difficulties [41,42]. Therefore, we must address the seriousness of gaming addiction and take effective interventions to help addicts recover a healthy state of life.

### 1.3. Inhibitory Control

Researchers have examined the processes by which gaming addiction leads to aggression, with inhibitory control being one of the key factors explaining this relationship. The Interaction of the Person-Affect-Cognition-Execution model [43] postulates that gaming addiction, along with other addictive behaviors, stem from interactions between susceptibility variables, affective and cognitive responses to specific stimuli, and impaired executive functioning. Argyriou et al. [44] revealed that individuals with gaming addiction were more prone to exhibit impaired response inhibition compared to healthy individuals through a meta-analysis. A laboratory study [45] involving 206 participants employed neuropsychological tests and clinical interviews found a significant increase in aggression as inhibitory control diminished. Similar effects have been demonstrated in multiple studies [4,46]. In sum, inhibitory control is negatively affected by gaming addiction and may subsequently trigger aggression [47].

### 1.4. Risk Preference

Theoretically, gaming addiction leads to an imbalance between two systems: the impulsive system based on emotions and the reflective system based on cognitive control [48,49]. By using a cross-temporal choice task that has been shown to predict a variety of addictive behaviors [50], Turel and He [51] found that the use of problematic social networks was associated with a preference for immediate gratification, which may be related to differences in the morphology of the insula cortex. High impulsivity promotes specific behaviors. For example, a preference for immediate gratification regardless of long-term negative consequences characteristic pattern of addictive behaviors [52], and a variety of aggressive behaviors may also occur simultaneously [53,54]. Thus, there is sufficient evidence that gaming addiction leads to an increase in aggressive behavior, with risky decision-making as a potential mediating effect.

Therefore, another key factor linking gaming addiction and aggression is risk preference. However, previous studies have not provided in-depth analyses of individuals’ risk preference behaviors, whereas the present study used a Bayesian hierarchical drift-diffusion model to interpret the risk preference task data. A key feature of gaming addiction is prolonged exposure to the intense rewards in video games, which can lead to a decrease in an individual’s sensitivity to natural rewards such as money and social praise [55]. The decision-making under risk, induced by altered reward sensitivity, may further lead to a range of aggressive behaviors in individuals, such as impulsivity [56] and cyberbullying [57]. Numerous studies have shown that individuals with gaming addiction exhibit a greater willingness to take risks when making decisions [54,58,59,60]. The risk preference, driven by reward sensitivity in individuals with gaming addiction can exacerbate a range of externalizing behaviors such as aggression [61], impulsivity [62], and substance abuse [59]. These findings offer compelling evidence that risk preference plays a mediation role in the path from gaming addiction to aggression.

### 1.5. Mediation Model

Inhibitory control and risk preference represent two distinct but related psychological traits in individual decision-making and behavior. A tripartite neurocognitive model suggests that the development and maintenance of gaming addiction is associated with failures of self-control, impulsivity, and dysfunction in reflective brain systems [31]. Research suggests that differences in inhibitory control may be related to the activity and connectivity of brain regions that are also involved in the neural processes of risk assessment and decision-making [63]. For example, the prefrontal cortex is also involved in the processing and integration of information during risky decision-making while performing inhibitory control functions [64]. Meanwhile, Laino Chiavegatti et al. [65] found that individuals with weaker inhibitory control tend to show higher risk preferences. Therefore, understanding the roles of inhibitory control and risk preference in the potential mediator model can contribute to a deeper understanding of the psychological mechanisms behind individual decisions and behaviors.

### 1.6. The Present Study

This study aimed to examine the relationship between gaming addiction and verbal aggression, with inhibitory control and risk preference examined as mediating variables. This paper aims to study the following aspects:

I. The effect of gaming addiction on verbal aggression.

II. The role of inhibitory control and risk preference in the path from gaming addiction to verbal aggression.

III. Explaining the selection process of risk preferences using Bayesian hierarchical drift-diffusion modeling.

Of the variables, verbal aggression and gaming addiction were measured by self-report questionnaires; Inhibitory control and risk preference were measured by behavioral experiments. A Bayesian hierarchical drift-diffusion model was employed to interpret the data from the risk preference task.

## 2. Methods

### 2.1. Participants

A total of 252 undergraduate students were recruited to participate in the study (mean age 19.60 years, SD: 1.44 years, range 17 to 29 years); 50.79% of the participants were women (mean age 19.48 years, SD: 1.46 years, range 17 to 27 years); 49.21% of the participants were men (mean age 19.73 years, SD: 1.42 years, range 17 to 29 years). Their average recreational time per day is shown in Table 1. All participants had normal or corrected-to-normal vision. Written informed consent was obtained from each participant before the experiment.

All participants were recruited through online adverts, posters, and word of mouth. The goal of this study is to explore the complexity and multiplicity of aggressive behaviors without focusing on a specific population. Therefore, inclusion criteria for participants included only normal vision or corrected vision, no history of psychiatric illness, and having received adequate sleep before participation in the experiment. Participants were informed that the data would be anonymized and that they had the right to withdraw from the experiment at any time. At the end of the experiment, they will receive 20 RMB as payment for the experiment. All materials and procedures were approved by the Human Research Ethics Committee of the university with which the first author was affiliated.

### 2.2. Research Instruments

#### 2.2.1. Questionnaires

The 20-item Internet Gaming Disorder Test developed by Pontes et al. [66] was administered before the commencement of the experiment to assess participants’ self-reported levels of gaming addiction. A video game time questionnaire was used for general information about the participants and the validity of the 20-item Internet Gaming Disorder Test measure. Additionally, the prosocial tendencies measure developed by Carlo et al. [67] was used to assess participants’ self-reported prosocial tendencies. This scale was used to corroborate the validity of the aggression questionnaire. Participants’ self-reported aggressive tendencies were assessed using the aggression questionnaire, which was developed by Buss and Perry et al. [68]. Details of the individual questionnaires can be found in the Supplementary Material.

#### 2.2.2. Antisaccade Task

The Antisaccade task, adapted from Miyake et al. [69], was employed to measure participants’ inhibitory control. Each trial of the Antisaccade task began with a fixation point in the center of the screen for a variable duration (every 250 ms, randomly selected between 1500 ms and 3500 ms). Subsequently, a visual cue (a solid black square) appeared on one side of the screen for 225 ms, which was followed by a target stimulus (a dashed box with an up or down arrow inside) on the other side of the screen for 150 ms, before being obscured by a gray cross-hatch. Participants were required to identify the direction of the arrows by pressing keys. Since the target stimuli were visible for only 150 ms, participants had to suppress the impulse to attend to the visual cue to identify the direction of the target stimuli accurately. Participants underwent 22 practice trials before completing 90 task trials. The proportion of correct responses in task trials was used as one of the indicators of inhibitory control.

#### 2.2.3. Go/No-Go Task

The Go/No-go task, adapted from Nosek et al. [70], was also administered to measure participants’ inhibitory control. In each trial, an asterisk serves as a fixation point in the middle of the screen for 400 ms. The letters “x” or “y” then appeared either alternately or repeatedly (twice in a row) at the same location for 1000 ms. The alternating appearance of letters was designated as a “Go” stimulus, requiring participants to respond by pressing the space bar as quickly as possible; while the repeated letters served as a “No-go” stimulus, necessitating participants to refrain from pressing the key. Participants completed 23 practice trials followed by 80 task trials. In the task trials, the ratio of “Go” to “No-go” was 17:3. The average accuracy of No-go trials was used as one of the indicators of inhibitory control.

#### 2.2.4. The Cup Task

The cup task [71] was used to test risk preference. The task was divided into gain and loss scenarios, each comprising 72 trials. Participants were required to decide between a fixed option (1 RMB) and one of the nine risky options. The risky options include three likelihoods of winning or losing (1/2, 1/3, or 1/5) plus three amounts (2, 3, or 5 RMB). These risky options are divided into three categories (risk-advantageous, risk-neutral, or risk-disadvantageous) according to whether the selection of risky options is profitable (winning more money or losing less money) or not. These three categories along with the two win or loss conditions comprised the six combination conditions, and the proportion of risky options selected in each combination condition was used in the formal analyses. In addition, the choices in each trial and the reaction times were also recorded for further computational modeling.

### 2.3. Procedure

Upon arrival, participants took a three-minute break before providing written informed consent. Participants were informed that their participation was voluntary and assured that their information and data would be anonymous and confidential. Participants were then instructed to complete the questionnaires in the following order: screen time questionnaire, prosocial tendencies measure, aggression questionnaire, 20-item Internet gaming disorder test, and Internet gaming disorder scale. On average, participants completed the questionnaires within 15 min. Following the questionnaires, participants proceeded to complete the Antisaccade task (approximately 10 min), the Go/No-go task (approximately 10 min), and the cup task (approximately 15 min) in order.

Participants in this study completed the questionnaires with an experimenter, and each questionnaire was confirmed to be free of missing values before proceeding to the next step, so there were no missing values. For the Antisaccade task and the Go/No-go task, participants would be removed if they were outside three standard deviations of accuracy, but no participants were found to meet this removal criterion.

### 2.4. Statistical Analysis

#### 2.4.1. Validity Analysis

A series of correlational analyses were used to validate the measures of gaming addiction and aggression: (1) Both indicators of gaming addiction were hypothesized to have positive correlations with average daily video game time; (2) Given that aggression and prosociality are treated as opposing psychological constructs, a negative correlation between the scores from the prosocial tendencies measure and the aggression questionnaire was anticipated.

#### 2.4.2. The Hierarchical Drift Diffusion Model

To delve deeper into the decision-making process in the cup task, a hierarchical drift-diffusion model [72] was used in analyzing the trial-level data of the cup task. The hierarchical drift-diffusion model constructs the decision-making process as an accumulation of noisy evidence, culminating in reaching one of two decision boundaries, signifying a decision. Using a Markov chain Monte Carlo method, the model estimates the joint posterior probability density distributions of all parameters and fits them accordingly [72]. The model consists of three primary parameters: *non-decision time* (*t*), which accounts for non-decision-making processes like perceptual encoding and motor execution; *drift rate* (*v*), indicating the speed of evidence accumulation towards the boundaries; *threshold* (*a*), representing the threshold between two decision boundaries.

Hierarchical Bayesian estimation was implemented using the Python toolkit HDDM 0.8.0 [72], running 4 independent Markov chains with 20,000 samples per chain and the first 2000 trials as burn-in trials. Parameters *a* and *v* were fitted in groups based on six combinations of conditions (risk-advantageous/risk-neutral/or risk-disadvantageous × gain/loss), and parameter *t* was fitted for gain or loss conditions separately. The model comprises 14 parameters in total. Table 2 presents the terms and explanations for each parameter. Model convergence was assessed by the Gelman-Rubin’s *R*-hat statistic [72], which indicates the ratio of the intra- and inter-chain variance in each iteration of the Markov chain, with values closer to 1 indicating that the individual chains are more likely to come from the same distribution.

#### 2.4.3. Mediation Model

Descriptive statistics and Pearson’s correlation analysis were used to characterize the sample and evaluate the interrelationships among the variables. In correlation analyses, we found that inhibitory control did not show a significant correlation with gaming addiction or aggression (or its sub-dimensions, refer to Table 3). Meanwhile, for the behavioral outcomes of the cup task and its model parameters, only the *v*_loss & disadvantage_ significantly correlated with both gaming addiction (*r* = −0.250, *p* < 0.001) and verbal aggression (*r* = −0.164, *p* = 0.009). No such relationship was found for the other sub-dimensions of aggression.

**Figure 1:**
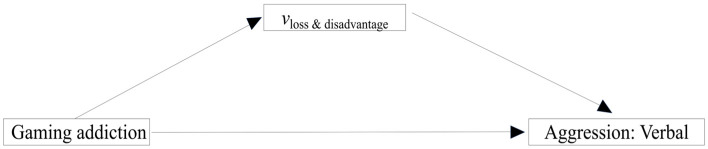
The hypothetical model. Note. The tested effects included: (1) the direct effect of gaming addiction to verbal aggression and (2) the mediation effect of drift rate in the loss risk disadvantage condition in the path from gaming addiction to verbal aggression. Vloss & disadvantage indicates the drift rates in the risk disadvantage condition when losing money is used as feedback. Gaming addiction: average scores of the 20-item Internet gaming disorder test. Aggression: Verbal is the one of sub-dimensions of the aggression questionnaire.

Therefore, this study analyzes only the mediating role of *v*_loss & disadvantage_ on the pathway from gaming addiction to verbal aggression. The full hypothesis model (Figure 1) included: (1) the direct effect of gaming addiction on verbal aggression; (2) the mediation effect of *v*_loss & disadvantage_ in the path from gaming addiction to verbal aggression. Given the potential impact of *a_loss & disadvantage_* and *t_loss_* on decision time, they were included as covariates in the analysis. Additionally, to ensure the independence of verbal aggression and to avoid confounding factors, the present study included other dimensions (physical aggression, angry aggression, hostility aggression, and self-aggression) of aggression as control variables. A bootstrapping procedure was used to estimate the indirect effect with a 95% confidence interval (CI). The indirect effect is deemed significant if zero is excluded from the 95% CI. Mediation models with the other dimensions of aggression as dependent variables were presented in the supplementary material.

## 3. Results

### 3.1. Correlational Analysis and the Cut-Off Point for Gaming Addiction

Table 3 and Table 4 show the results of the correlational analysis. Gaming addiction was significantly and positively correlated with physical aggression (*r* = 0.201, *p* < 0.001), anger aggression (*r* = 0.156, *p* = 0.013), verbal aggression (*r* = 0.157, *p* = 0.005), hostile aggression (*r* = 0.221, *p* < 0.001), and self-aggression (*r* = 0.257, *p* < 0.001). There was no significant correlation between game addiction and the outcomes of *Go/No-go task* (*r* = 0.034, *p* = 0.588) and the *Antisaccade task* (*r* = 0.076, *p* = 0.227). Additionally, *v_loss & disadvantage_* correlated with both gaming addiction (*r* = −0.250, *p* < 0.001) and verbal aggression (*r* = −0.164, *p* = 0.009). All dimensions of the 20-item Internet Gaming Disorder Test were significantly correlated with physical aggression (*r*s > 0.148, *p*s < 0.018). Except for mood, all other dimensions were significantly correlated with verbal aggression (*r*s > 0.103, *p*s < 0.045). Indicating that the mood dimension may have less impact in predicting verbal aggression.

Linear regression analyses were performed on game addiction (X) with *v_loss & disadvantage_* (Y1) and verbal aggression (Y2) as dependent variables. The results are Y1 = −0.018X − 0.337, and Y2 = 0.044X + 9.708, indicating that when scores on the 20-item Internet Gaming Disorder Test reached 42.16 and 42.97, respectively, *v*_loss & disadvantage_ (−1.096) and verbal aggression (11.599) reach their mean value. In other words, individuals were at higher risk in terms of *v*_loss & disadvantage,_ and verbal aggression if their scores on the 20-item Internet Gaming Disorder Test exceeded 42. Therefore, 42 could be considered as the cut-off point for the 20-item Internet Gaming Disorder Test. Based on this cut-off point, 113 participants in this study are at higher risk for gaming addiction (57.52% male, mean age 19.65 years, SD: 1.08 years, range 18 to 24 years).

### 3.2. Validity Analysis

Table 3 and Table 4 show the descriptive statistics and the results of the correlational analyses. For the validity of the measures: (1) Average daily video game time was significantly and positively related to scores from the 20-item Internet gaming disorder test (*r* = 0.528, *p* < 0.001); (2) A significant negative correlation was observed between the overall score of the aggression questionnaire and the prosocial tendencies measure (*r* = −0.162, *p* = 0.010). The results provide evidence for the measurement validity of the 20-item Internet gaming disorder test and the aggression questionnaire.

### 3.3. Hierarchical Drift Diffusion Model

**Figure 2:**
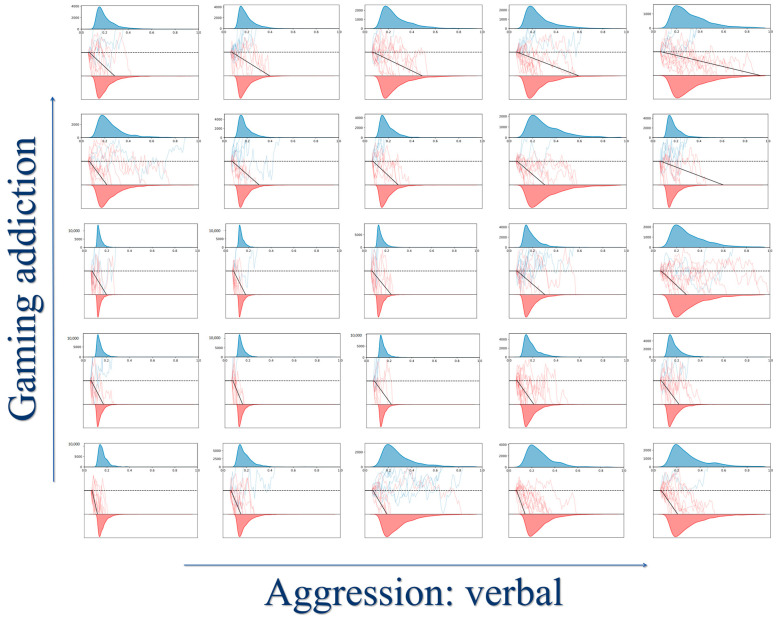
Hierarchical drift-diffusion modeling based on cup tasks. *Note.* The effects of aggression: verbal (*X*-axis) and gaming addiction (*Y*-axis) on the drift rate (solid black line in the middle of each model) are modeled in our decision space. According to the correlation matrix analysis, the drift rate gradually decreases (from left to right) as verbal aggression increases, while the drift rate gradually decreases (from bottom to top) as gaming addiction increases. *Gaming addiction*: average scores of the 20-item Internet gaming disorder test. *Aggression: Verbal* is the one of sub-dimensions of the aggression questionnaire. The blue colour in the diagram represents the non-risk response boundary and the red colour represents the risk response boundary. Plot inspired by Saleh et al. [73].

The hierarchical drift-diffusion model reached convergence with Gelman-Rubi ^^^*R* < 1.001. Indicating that the model fits the data well. The descriptive statistics for each parameter are presented in Table 5. It can be seen that in both the win condition and the loss condition when the mathematical expectation of choosing the risky option is higher, the drift rate of the participants is positive. Indicating that they accumulate selection tendency in the direction of choosing the risky option when the risky option is profitable. Similarly, when the risky option is unprofitable, the drift rate is negative, and participants accumulate selection tendencies toward the fixed option. According to the correlation matrix (Table 4), *v*_loss & disadvantage_ correlated with both gaming addiction (*r* = −0.250, *p* < 0.001) and verbal aggression (*r* = −0.164, *p* = 0.009). It decreases as gaming addiction and verbal aggression increase (refer to Figure 2).

In the cup task, *threshold (a)* indicates the amount of cumulative evidence needed to make the decision. A lower *threshold* indicates that individuals are more likely to decide with litter evidence, while a higher *threshold* indicates that individuals need more evidence to make a decision. The results of this study show that individuals have the lowest average *threshold* in gain and risk-advantaged conditions. The *drift rate (v)* indicates the rate at which evidence accumulates during the decision process. For risk-favorable options, individuals may have a higher *drift rate*, meaning that they accumulate positive decision evidence more quickly. Conversely, for risk-unfavorable options, the *drift rate* may be lower because they need more negative evidence to change their decision. The results of this study suggest that, on average, individuals accumulate evidence fastest in the loss and risk-neutral conditions. *Non-decision time (t)* represents the time spent in the non-decision process other than the decision itself, such as time spent attending to options and moving fingers to response keys. The results of this study indicate that individuals had the shortest average non-decision time in the loss condition.

### 3.4. Mediation Model

**Figure 3:**
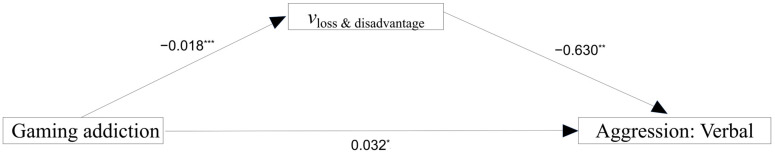
Path coefficient diagram for the mediation model. Note. *, ** and *** indicates *p* < 0.05, *p* < 0.01 and 0.001, respectively. Standardized path coefficients for the mediating model. *V_loss & disadvantage_* indicates the drift rates in the risk disadvantage condition when losing money is used as feedback. *Gaming addiction*: average scores of the 20-item Internet gaming disorder test. *Aggression: Verbal* is the one of sub-dimensions of the aggression questionnaire.

For the mediation model, the direct effect of gaming addiction on verbal aggression did not reach a significant level (*β* = 0.005, *SE* = 0.012, *p* = 0.684, refer to Figure 3). However, the indirect path from gaming addiction to verbal aggression through *v*_loss & disadvantage_ was significant (indirect effect = 0.008, *p* = 0.027, 95% CI = [0.002, 0.017]), indicating that the *v*_loss & disadvantage_ fully mediates the relationship between gaming addiction and verbal aggression. More gaming addiction predicts less drift rate in loss and risk-disadvantage condition, *β* = −0.018, *SE* = 0.004, *p* < 0.001, and a larger *v*_loss & disadvantage_ negatively predicts verbal aggression, *β* = −0.454, *SE* = 0.175, *p* = 0.009.

## 4. Discussion

Based on the results of correlational analysis, several noteworthy correlations between gaming addiction and various forms of aggression have been identified. Gaming addiction shows significant positive correlations with physical aggression, anger aggression, verbal aggression, hostile aggression, and self-aggression. These findings suggest that individuals who exhibit higher levels of gaming addiction are more likely to engage in these aggressive behaviors. This aligns with previous research indicating a link between excessive gaming and increased aggression [30]. However, this study used a cross-sectional design that only reveals correlations and does not directly infer a causal relationship between gaming addiction and verbal aggression.

Gaming addicts are more likely to exhibit verbal aggression, which is consistent with previous findings on general aggression [14,15,16]. Video games, social platforms, video websites, and other similar forms of media may exhibit or encourage uncivilized or unethical verbal behaviors [74,75], which might exert an osmotic effect on individuals who frequently utilize the Internet [12], thereby reinforcing individuals’ tendencies towards verbal aggression. Compared to other forms of aggression, verbal aggression is at a low cost and threshold for implementation. Verbal aggression is more difficult to detect than physical aggression and is often more enduring and profound [34]. This might account for the increasing prevalence of verbal aggression in contemporary times [76].

### 4.1. Mediation Model

The findings of the mediation model revealed that the *v*_loss & disadvantage_ fully mediated the relationship between gaming addiction and verbal aggression. However, the direction of the paths differs from our expectations. According to the introduction, individuals with higher levels of gaming addiction have a greater risk preference and therefore will accumulate a selection tendency towards the risky option even though the risky option (compared to the fixed option) has a smaller expected profit and is more likely to result in a loss. Contrariwise, in the current results, gaming addiction was negatively (but not positively) correlated with *v*_loss & disadvantage_, indicating that gaming addicts performed better at avoiding losses in unfavorable conditions. This drift rate was also negatively correlated with verbal aggression, meaning that individuals with less risk preference tend to perform more verbal aggression. In sum, lower but not higher risk preferences fully mediated the path from gaming addiction to verbal aggression.

One explanation is that gaming addicts become less sensitive to natural rewards or losses. In games, repeated high-arousal stimuli and rewards can physiologically impair an individual’s decision-making processes [59], making the individual less sensitive to natural rewards and risks [77]. Therefore, gaming addicts may only show a risk preference for game-related cues. They are less sensitive to whether they win or lose money in the cup task because the cue (i.e., cups) in this task is neutral and not game-related. This lack of sensitivity to natural loss may lead them to poorer empathy [78] and therefore be more likely to display verbal aggression.

Another explanation is that gaming addicts attempt to escape adverse situations or minimize losses through fast decision-making. Gaming addiction sensitizes individuals to higher levels of punishment [68], and forces them to make quicker decisions to gain rewards or avoid losses [79]. This concern may be particularly pronounced in terms of their financial and temporal losses [80,81]. As a result, in the loss and unfavorable conditions, they accumulate selection tendencies towards the fixed option faster, as indicated by a smaller *v*_loss & disadvantage_. This impulsive choice to avoid a loss may further increase a variety of aggressiveness, including verbal aggression [4].

The results from the mediation model indicate that individuals with a preference for low risk showed a higher propensity for verbal aggression. This may reflect some alteration or deficiency in the decision-making process of gaming addicts in the face of loss and risk, such as the individual’s different choice of emotional regulation and coping strategies [82]. When individuals perceive lower natural rewards or losses, they may lack the resources to effectively cope with negative emotions, leading to more frequent resort to verbal aggression to release stress or express dissatisfaction. Additionally, gaming addiction may further increase the likelihood of verbal aggression by decreasing their psychological flexibility in coping with stress [83]. This finding highlighted the importance of mental health interventions. By enhancing an individual’s ability to regulate their emotions and diversify their coping strategies, the tendency for individuals to resort to verbal aggression in the face of negative emotions is reduced. The potential mediating mechanisms suggested above all need to be explored in depth by further research in the future.

The results of the model with other dimensions of aggression as dependent variables showed that *v*_loss & disadvantage_ did not mediate the relationship between gaming addiction and other aggression. This is the same as our expectation that the relationship between gaming addiction and aggression is not generalized but requires a specific analysis of the form of aggression. Due to the specificity of verbal aggression [33], more forms of post-game aggression are now shifted to verbal aggression. This suggests that there is no direct role relationship between gaming addiction and other forms of aggression. Gaming addiction does not directly cause the likelihood that individuals will engage in other forms of actual aggressive behavior. This result also warns us that more attention needs to be paid to the mental health of individuals with gaming addiction to reduce the development of verbal aggression.

The applicability of the findings is influenced by several factors, including different types of games and their varying effects on individuals with gaming addictions. Different types of games and gaming habits may have different effects on verbal aggression. Therefore, the findings of this study may not apply to all individuals with gaming addiction and may not generalize to all types of games. Future research could further explore these differences to deepen our understanding of gaming contexts and their potential impact on verbal aggression.

### 4.2. Risk Preference

The results of the correlation analysis showed that gaming addiction showed more risk behaviors under unfavorable conditions of loss and risk. According to prospect theory [84], people are more willing to avoid losses than to gain gains of equal value. This may be due to the fact that gaming addicts are particularly sensitive to losses [68], leading them to be more cautious and conservative to avoid any possible losses, even if it means giving up opportunities for possible gains. This may also be because prolonged addiction to gaming may lead to a diminished perception of social rewards for the individual. Making them more inclined to avoid possible negative outcomes rather than pursuing potential positive gains.

However, under other conditions, gaming addiction individuals did not show significant differences in their choices. This may be because the cup task used in this study belongs to the objective risk domain rather than the ambiguous domain (which does not directly provide risk information) [58]. Adverse decision making under objective risk indicates an increased propensity for risk-taking [85], whereas adverse decision making under ambiguity is more reflective of deficits in affective-behavioral regulation [86]. Therefore, the results of the present study suggest that the risk-taking tendencies of individuals with gaming addiction in winning situations are not significantly different from those of normal individuals. However, this study lacked the exploration of emotional behavior in game-addicted individuals.

### 4.3. Inhibitory Control

For inhibitory control, contrary to previous findings [16,60,87,88,89], inhibitory control was not significantly correlated with either gaming addiction or aggression. Insufficient measurement validity of the inhibitory control task is one potential reason, but we do not believe this reason is substantiated. Two inhibitory control tasks were used in the study, one by attentional inhibition (Antisaccade task) and the other by motor inhibition (Go/No-go task). The significant correlation between the performances of the two inhibition tasks suggests that they measure the same constructs and can corroborate the validity of each other. In addition, these two tasks are common for measuring inhibitory control in research on executive function [82] or cognitive control [90,91]. Therefore, there is ample reason to believe that the measurement of inhibitory control in this study is valid.

Another potential reason is that neutral cues were used in both inhibitory control tasks, but gaming addicts may be more sensitive to gaming-related cues. Previous studies [92,93] have demonstrated a link between individuals with gaming addiction and inhibitory control measured by the Go/No-go task when the cues are related to video games, as opposed to neutral cues. Similar results have been found on social media addiction [94], where social media-addicted individuals are more likely to show diminished inhibitory control when confronted with social media-related cues in the Go/No-go task. Therefore, inhibition deficits in individuals with gaming addiction might be cue-dependent and may not generalize to neutral cues.

### 4.4. Contribution of the Present Study

One major contribution of this study is the finding that inhibitory control does not play a significant role in gaming addiction and aggression among college students. Previous studies on adolescents’ gaming addiction point to imbalances in neurological development as a key factor [95]. Over-rapid development of the limbic system, which is associated with reward processing, and delayed development of the prefrontal lobe, which is associated with cognitive control [96,97,98], are the main reasons why gaming addiction leads to a variety of problematic behaviors. However, neither gaming addiction nor aggression were significantly associated with impaired inhibitory control in this study. It can be assumed that by college age, the neural basis of inhibitory control has matured and is no longer a critical factor in mediating the pathway from gaming addiction to aggression.

Another major contribution is the confirmation of the mediating role of *v*_loss & disadvantage_ in the path from gaming addiction to verbal aggression, emphasizing the need for a more deliberate design of reward mechanisms in games. Game designers tend to enhance the game’s appeal and enjoyment by integrating uncertain rewarding elements like randomly dropped golds and items [99,100]. This design may contribute to reduced sensitivity to natural rewards and increased loss-aversion bias, which plays a key role in linking gaming addiction to verbal aggression [100,101,102,103]. Therefore, if more studies parameterize the process of acquiring risk preferences in games by using computational modeling, it could help game designers to more precisely regulate the extent of rewards in games to avoid further behavioral problems like verbal aggression.

### 4.5. Limitations

First, ceiling effects were observed in the Go/No-go task and the antisaccade task, complicating the interpretation of the path from gaming addiction to inhibitory control. However, as mentioned earlier, the sample in this study was college students, whose inhibitory control systems are mature (unlike adolescents, who are in the developmental stage). Therefore, this result is by the real situation. Also, this study was cross-sectional and could not determine a causal relationship between gaming addiction and verbal aggression. A longitudinal study is needed to further determine the causal relationship between the two. Second, the data on gaming addiction and aggression in this study came from self-reported measures, which could be biased or misinterpreted. More accurate data could be provided in the future through objective measurements or observations. Last, a single task was used in the study to examine risk preference. The use of different types of risk preference tasks can corroborate the validity of the measurements and increase the reliability of the conclusions.

## 5. Conclusions

This paper reveals the relationship between gaming addiction and verbal aggression and its underlying processes. It was found that gaming addiction may lead to reduced sensitivity to natural rewards and excessive loss avoidance, which in turn increases the likelihood that individuals will tend towards verbal aggression. This provides important empirical support for social psychology and media influence research and contributes to further understanding of the formation and expansion of verbal aggression in modern society. At the same time, this study did not find a significant association between inhibitory control and aggression in gaming addiction, but this finding is also significant. This suggests that the development of inhibitory control systems may no longer be a key factor in explaining the effects of gaming addiction on behavior in adulthood, which contrasts with the findings of previous studies on adolescents. Finally, this study points to the importance of reward mechanisms in game design. By more accurately designing reward mechanisms, game addicts may be able to reduce their decreased sensitivity to natural rewards and excessive avoidance of losses, thereby reducing behavioral patterns that may lead to problems such as verbal aggression.

## Figures and Tables

**Table 1:** Descriptive statistics of participants’ various recreational time.

*Recreation Program*	*M*	*SD*
*Video game*	59.29	70.77
*TV*	40.96	48.10
*Short video*	78.02	72.62
*Card game*	4.61	17.68
*Text-based media*	38.77	48.52
*Webcast*	7.00	25.30

Note. The table presents the average daily time participants spent using each recreational program. Time is measured in minutes. *N* = 252.

**Table 2:** Explanations of the terms in the hierarchical drift-diffusion model.

Terms	Explanations
*a*_win & advantage_	The threshold in the risk advantage condition when winning money is used as feedback.
*a_win_*_& disadvantage_	The threshold in the risk disadvantage condition when winning money is used as feedback.
*a_win_*_& neutral_	The threshold in the neutral condition when winning money is used as feedback.
*a*_loss & advantage_	The threshold in the risk advantage condition when losing money is used as feedback.
*a*_loss & disadvantage_	The threshold in the risk disadvantage condition when losing money is used as feedback.
*a*_loss & neutral_	The threshold in the neutral condition when losing money is used as feedback.
*v*_win & advantage_	The drift rates in the risk advantage condition when winning money are used as feedback.
*v_win_*_& disadvantage_	The drift rates in the risk disadvantage condition when winning money are used as feedback.
*v_win_*_& neutral_	The drift rates in the neutral condition when winning money are used as feedback.
*v*_loss & advantage_	The drift rates in the risk advantage condition when losing money are used as feedback.
*v*_loss & disadvantage_	The drift rates in the risk disadvantage condition when losing money are used as feedback.
*v*_loss & neutral_	The drift rates in the neutral condition when losing money are used as feedback.
*t*_win_	The non-decision time when winning money is used as feedback.
*t*_loss_	The non-decision time when losing money is used as feedback.

*Note.* The hierarchical drift-diffusion model contains a total of 14 parameters. Positive values of the drift rate represent an accumulation of choice propensities towards the risky option, while negative values represent an accumulation of choice propensities towards the fixed option. The absolute value of the drift rate represents the rate at which the propensity to a certain choice accumulates.

**Table 3:** Means, standard deviations, and correlations of the variables.

*Variable*	*M*	*SD*	1	2	3	4	5	6	7	8	9	10	11	12	13	14	15	16	17	18	19	20	21	22
*1. Gaming addiction*	42.595	13.131	1.000																					
*2. Prosocial tendencies measure*	100.754	11.558	−0.031	1.000																				
*3. Aggression: Physical*	13.627	4.318	0.201 **	−0.075	1.000																			
*4. Aggression: Verbal*	11.599	3.332	0.175 **	−0.054	0.499 ***	1.000																		
*5. Aggression: Anger*	13.643	5.063	0.156 *	−0.107	0.488 ***	0.638 ***	1.000																	
*6. Aggression: Hostility*	16.837	4.986	0.221 ***	−0.261 ***	0.373 ***	0.412 ***	0.578 ***	1.000																
*7. Aggression: Self-aggression*	9.996	3.981	0.248 ***	−0.098	0.421 ***	0.360 ***	0.598 ***	0.603 ***	1.000															
*8. Overall score of Aggression*	65.702	16.863	0.257 ***	−0.162 **	0.711 ***	0.724 ***	0.864 ***	0.789 ***	0.773 ***	1.000														
*9. Antisaccade task*	0.939	0.076	0.076	0.015	−0.031	0.043	0.060	−0.015	−0.014	0.011	1.000													
*10. Go/No-go task*	0.779	0.188	0.034	0.006	0.021	−0.004	0.028	0.044	0.017	0.030	0.321 ***	1.000												
*11.%Choice_win & advantage_*	0.735	0.223	0.039	0.023	0.051	0.106	−0.015	−0.036	−0.048	0.007	0.014	0.092	1.000											
*12.%Choice_win & disadvantage_*	0.070	0.165	−0.048	0.108	−0.020	−0.116	−0.101	−0.081	0.045	−0.072	0.062	−0.016	0.118	1.000										
*13.%Choice_win & neutral_*	0.275	0.242	−0.005	0.098	0.041	0.004	−0.017	0.011	0.004	0.011	0.063	0.059	0.468 ***	0.582 ***	1.000									
*14.%Choice_loss & advantage_*	0.874	0.187	0.005	0.014	0.055	0.072	−0.006	−0.044	−0.096	−0.009	0.008	0.049	0.349 ***	−0.236 ***	0.013	1.000								
*15.%Choice_loss & disadvantage_*	0.168	0.216	−0.140 *	−0.002	0.013	−0.117	−0.052	0.002	0.073	−0.018	−0.098	−0.149 *	−0.126 *	0.319 ***	0.185 **	0.182 **	1.000							
*16.%Choice_loss & neutral_*	0.589	0.286	−0.112	−0.016	0.015	−0.059	−0.008	−0.002	−0.011	−0.013	−0.016	0.038	0.159 *	0.030	0.164 **	0.624 ***	0.509 ***	1.000						
*17.Gaming addiction: Salience*	6.119	2.869	0.893 ***	0.024	0.218 ***	0.154 **	0.123	0.180 **	0.181 **	0.219 ***	0.079	0.052	−0.016	−0.026	−0.016	−0.010	−0.116	−0.0127 *	1.000					
*18.Gaming addiction: Mood*	8.786	2.340	0.473 ***	−0.025	0.163 **	0.103	0.021	0.154 *	0.153 *	0.150 *	−0.003	0.050	0.048	−0.001	0.038	−0.017	−0.043	−0.059	0.432 ***	1.000				
*19.Gaming addiction: Tolerance*	6.623	2.745	0.845 ***	0.019	0.148*	0.126*	0.107	0.170 **	0.203 **	193**	0.072	0.026	0.036	−0.026	0.007	0.049	−0.094	−0.048	0.771 ***	0.389 ***	1.000			
*20.Gaming addiction: Withdrawal*	5.254	2.249	0.838 ***	−0.091	0.231 ***	0.167 ***	0.106 *	0.245 ***	0.244 ***	270 ***	0.059	−0.020	−0.006	−0.017	−0.053	0.007	−0.072	−0.082	0.673 ***	0.368 ***	0.643 ***	1.000		
*21.Gaming addiction: Conflict*	10.190	2.853	0.703 ***	−0.003	0.163 **	0.203 **	0.109	0.157 *	0.210 ***	0.210 ***	0.023	−0.062	0.023	−0.012	−30.774 × 10^−4^	0.006	−5.784 × 10^−4^	−0.082	0.631 ***	0.393 ***	0.573 ***	0.644 ***	1.000	
*22.Gaming addiction: Relapse*	5.774	2.802	0.835 ***	0.025	0.165 **	0.149 *	0.156 *	0.135 *	0.220 **	210 ***	0.033	0.019	0.079	−0.009	0.068	0.010	−0.099	−0.114	0.727 ***	0.347 ***	0.641 ***	0.643 ***	0.579 ***	1.000

Note. N = 252. This table reports the mean (M) and standard deviation (SD) of each questionnaire and behavioral indicator, as well as the results of the correlational analyses. Gaming addiction: average scores of the 20-item Internet gaming disorder test. Prosocial tendencies measure: average scores of the prosocial tendencies measure. Aggression: Physical, Aggression: verbal, Aggression: anger, Aggression: hostility, and Aggression: self-aggression are the average scores of the six sub-dimensions of the aggression questionnaire, respectively. Antisaccade task: the proportion of correct responses in the task. Go/No-go task: the average accuracy of “no-go” trials in the task. %Choice_win & advantage_, %Choice_win & disadvantage_, and %Choice_win & neutral_: percentage of risky options in risk advantage, disadvantage, and neutral conditions, respectively, when winning money is used as feedback. %Choice_loss & advantage_, %Choice_loss & disadvantage_, and %Choice_loss & neutral_: percentage of risky options in risk advantage, disadvantage, and neutral conditions, respectively, when losing money is used as feedback. Gaming addiction: Salience, Gaming addiction: Mood, Gaming addiction: Tolerance, Gaming addiction: Withdrawal, Gaming addiction: Conflict, Gaming addiction: Relapse are the six sub-dimensions of the 20-item Internet Gaming Disorder Test. *, **, and *** indicates *p* < 0.05, 0.01 and 0.001, respectively.

**Table 4:** Correlations between the parameters of the cup task and the other variables.

*Variable*	*11.a*_win & advantage_	*12.a*_win & disadvantage_	*13.a*_win & neutral_	*14.v*_win & advantage_	*15.v*_win & disadvantage_	*16.v*_win & neutral_	*17.t*_win_	*18.a*_loss & advantage_	*19.a*_loss & disadvantage_	*20.a*_loss & neutral_	*21.v*_loss & advantage_	*22.v*_loss & disadvantage_	*23.v*_loss & neutral_	*24.t*_loss_
*1. Gaming addiction*	−0.042	−0.053	−0.022	0.041	−0.108	−0.052	−0.009	−0.076	0.004	−0.058	0.025	−0.25 ***	−0.106	−0.037
*2. Antisaccade task*	−0.033	−0.054	0.037	0.024	0.054	0.052	−0.008	−0.018	0.059	0.038	0.025	−0.078	−0.022	−0.023
*3. Go/No-go task*	0.076	0.05	0.097	0.059	0.009	0.047	0.091	0.091	0.191 **	0.142 *	−0.029	−0.125 *	−0.01	0.143 *
*4. Prosocial tendencies measure*	−0.114	−0.089	−0.116	0.046	0.062	0.091	−0.105	−0.152 *	−0.125 *	−0.168 **	0.055	−0.002	0.029	−0.035
*5. Aggression: Physical*	−0.099	−0.115	−0.123	0.04	−0.105	−0.016	−0.028	−0.08	−0.062	−0.094	0.075	−0.052	0.024	−0.082
*6. Aggression: Verbal*	−0.016	−0.056	−0.046	0.088	−0.126 *	−0.015	−0.004	−0.034	−0.026	−0.049	0.067	−0.164 *	−0.074	−0.129 *
*7. Aggression: Anger*	−0.033	−0.047	−0.038	−0.024	−0.130 *	−0.052	0.024	−0.049	−0.031	−0.038	−0.011	−0.086	−0.048	−0.045
*8. Aggression: Hostility*	0.019	0.031	−0.009	−0.043	−0.136 *	−0.03	0.015	−0.006	−0.008	−0.022	−0.033	−0.091	−0.035	−0.007
*9. Aggression: Self-aggression*	−0.064	−0.093	−0.08	−0.038	−0.041	−0.026	0.04	−0.139 *	−0.108	−0.121	−0.037	−0.03	−0.016	−0.019
*10. Overall score of Aggression*	−0.048	−0.067	−0.074	−0.001	−0.141 *	−0.038	0.013	−0.076	−0.058	−0.08	0.01	−0.105	−0.037	−0.066

Note. N = 252. This table reports correlations between the parameters of the cup task and the other variables. Gaming addiction: average scores of the 20-item Internet gaming disorder test. Prosocial tendencies measure: average scores of the prosocial tendencies measure. Aggression: Physical, Aggression: verbal, Aggression: anger, Aggression: hostility, and Aggression: self-aggression are the average scores of the six sub-dimensions of the aggression questionnaire, respectively. Antisaccade task: the proportion of correct responses in the task. Go/No-go task: the average accuracy of “no-go” trials in the task. The explanation of the parameters of the cup task is given in Table 2. *, **, and *** indicate *p* < 0.05, 0.01, and 0.001, respectively.

**Table 5:** Descriptive statistics of the parameters in the hierarchical drift-diffusion model.

Variable	*M*	*SD*
*a*_win & advantage_	1.783	0.030
*a*_win & disadvantage_	1.954	0.033
*a*_win & neutral_	1.812	0.030
*a*_loss & advantage_	2.203	0.043
*a*_loss & disadvantage_	2.454	0.044
*a*_loss & neutral_	2.178	0.041
*v*_win & advantage_	0.842	0.070
*v*_win & disadvantage_	−1.959	0.072
*v*_win & neutral_	−0.810	0.070
*v*_loss & advantage_	1.347	0.065
*v*_loss & disadvantage_	−1.095	0.064
*v*_loss & neutral_	0.276	0.063
*t*_win_	0.430	0.007
*t*_loss_	0.413	0.008

*Note.* The final hierarchical drift-diffusion model contains a total of 14 parameters. An explanation of the terms in the hierarchical drift-diffusion model is shown in Table 2. *N* = 252.

## Data Availability

The data sets used in this study are available from the corresponding author on reasonable request.

## Supplementary Materials

The following supporting information can be downloaded at: https://www.mdpi.com/article/10.3390/bs14080699/s1. References [66,67,104,105,106] are cited in Supplementary Materials.
