# Supplementary material for: When Games Influence Words: Gaming Addiction among College Students Increases Verbal Aggression through Risk-Biased Drifting in Decision-Making
Source: Behav Sci (Basel). 2024 Aug 11;14(8):699. doi: 10.3390/bs14080699 (PMC11352047; doi:10.3390/bs14080699)
Supplement: Supplementary file 1 [file behavsci-14-00699-s001.zip › behavsci-3117998-supplementary.pdf]

Supplementary material

1. Questionnaires:

1.1. Twenty-item Internet Gaming Disorder Test

The 20-item Internet Gaming Disorder Test developed by Pontes et al. [66] was administered prior to the commencement of the experiment to assess participants' self-reported levels of gaming addiction. A revised Chinese version of Qin et al. [104] was used in this study. It consists of 20 items with six factors: salience (e.g., "I often lose sleep because of long gaming sessions."), mood modification (e.g., "I play games to help me cope with any bad feelings I might have."), tolerance (e.g., " I have significantly increased the amount of time I play games over last year."), withdrawal (e.g., "When I am not gaming, I feel more irritable."), conflict (e.g., "I have lost interest in other hobbies because of my gaming."), and relapse (e.g., "I would like to cut down my gaming time but it's difficult to do."). The scale consists of 20 items and is scored using a five-point scale (5 = strongly agree, 1 = strongly disagree). The scores across the 20 items were averaged to create a composite score, with a higher composite score indicating more severe gaming addiction. Qin et al. [104] tested the reliability of this scale among Chinese university students. The Cronbach's alpha coefficient of the scale was 0.89 in the previous study, and 0.91 in the present study.

1.2. Video game time questionnaire

A questionnaire was also used in this study to support the validity of the gaming addiction measures by investigating the average daily screen time participants spent playing video games. The questionnaire contains four items: "The average number of days you play video games from Monday to Friday ", "The average number of days you play video games on Saturdays and Sundays", "Monday to Friday, on the days you play video games, what is the average time (in minutes) spent playing video games per day?" and "Saturdays and Sundays, on the days you play video games, what is the average time (in minutes) spent playing video games per day?". Total weekly game time can be calculated by: number of days spent playing video games on weekdays × average weekday game time + number of days spent playing games on weekends × average weekend game time. This total weekly game time was divided by 7 to create an indicator of average daily video game time (in minutes).

1.3. Prosocial tendencies measure

Prior to the beginning of the experiment, the prosocial tendencies measure developed by Carlo et al. [67] was used to assess participants' self-reported prosocial tendencies. A revised Chinese version of Kou et al. [105] was used in this study. This scale was use to corroborate the validity of the aggression. The scale consists of 26 items with six dimensions: public (e.g., "I can help others best when people are watching me"), anonymous (e.g., "I prefer to donate money anonymously"), dire (e.g., "I get the most

out of helping others when it is done in front of others."), emotional (e.g., "It is most fulfilling to me when I can comfort someone who is very distressed."), compliant (e.g., "When other people are around, it is easier for me to help needy others."), and altruism (e.g., "I believe that donating goods or money works best when it is tax-deductible."). Participants rated on a 5-point scale (1 = very nonconforming, 5 = very conforming), with a higher average score indicating more tendencies towards prosocial behaviors. The Cronbach's alpha of the prosocial tendencies measure was 0.91.

1.4. Aggression questionnaire

Participants' self-reported aggressive tendencies were assessed using the aggression questionnaire, which developed by Buss and Perry et al. [67]. A revised Chinese version of Li et al., [106] was used in this study. It consists of 30 items with five subscales: physical aggression (e.g., "Once in a while I can't control the urge to strike another person."), verbal aggression (e.g., "I tell my friends openly when I disagree with them."), anger (e.g., "I flare up quickly but get over it quickly."), hostility (e.g., "I am sometimes eaten up with jealousy.") and self-aggression (e.g., "I think of hurting myself when I am very irritable." and "I get hurt by carelessness when I am very angry."). Participants responded on a five-point scale (1 = not compliant, 5 = fully compliant), with higher scores indicating higher level of aggression. In the current study, the scores of the subscales were also used in the formal analysis. In previous studies, the Cronbach's alpha of the five sub-dimension scores of this questionnaire and the total score of the scale ranged from 0.60 to 0.89 [106]. The Cronbach's alpha of the five subscales ranged from .72 to .86, and that of the total score was 0.91 in present study.

2. Mediation model

The present study only analyzed the mediating role of $v_{\text{loss \& disadvantage}}$ in the path from game addiction to other aggression (physical aggression, angry aggression, hostility aggression, and self-aggression). The full hypothesis model included: (1) the direct effect of gaming addiction on other aggression; (2) the mediation effect of $v_{\text{loss \& disadvantage}}$ in the path from gaming addiction to other aggression. Given the potential impact of $a_{\text{loss \& disadvantage}}$ and t_{loss} on decision time, they were included as covariates in the analysis. Additionally, to ensure the independence of each aggression and to avoid confounding factors, the present study included other dimensions of aggression as control variables. A bootstrapping procedure was used to estimate the indirect effect with a 95% confidence interval (CI). The indirect effect is deemed significant if zero is excluded from the 95% CI.

2.1 Physical aggression

For the mediation model, the direct effect of gaming addiction on physical aggression did not reach the significant level (β = 0.026, *SE* = 0.018, p = 0.153). The indirect path from gaming addiction to physical aggression through $v_{\text{loss \& disadvantage}}$ was not significant (indirect effect = -0.003, p = 0.561, 95% CI = [-0.013, 0.005]), indicating that the $v_{\text{loss \& disadvantage}}$ does not mediates the relationship between gaming addiction and physical aggression. More gaming addiction predicts less drift rate in loss and risk-disadvantage condition, β = -0.017, *SE* = 0.004, p < 0.001, and a larger $v_{\text{loss \& disadvantage}}$ positively predicts verbal aggression, β = 0.0.153, *SE* = 0.261, p = 0.0557.

2.2 Anger aggression

For the mediation model, the direct effect of gaming addiction on anger aggression did not reach the significant level (β = -0.020, *SE* = 0.017, *p* = 0.231). The indirect path from gaming addiction to anger aggression through $v_{\text{loss \& disadvantage}}$ was not significant (indirect effect = $-8.194*10^{-4}$, *p* = 0.847, 95% CI = [-0.007, 0.007]), indicating that the $v_{\text{loss \& disadvantage}}$ does not mediates the relationship between gaming addiction and anger aggression. More gaming addiction predicts less drift rate in loss and risk-disadvantage condition, β = -0.018, *SE* = 0.004, $p < 0.001$, and a larger $v_{\text{loss \& disadvantage}}$ negatively predicts verbal aggression, β = 0.046, *SE* = 0.238, *p* = 0.847.

2.3 Hostility aggression

For the mediation model, the direct effect of gaming addiction on hostility aggression did not reach the significant level (β = 0.023, *SE* = 0.019, *p* = 0.237). The indirect path from gaming addiction to hostility aggression through $v_{\text{loss \& disadvantage}}$ was not significant (indirect effect = 0.002, *p* = 0.731, 95% CI = [0.002, 0.017]), indicating that the $v_{\text{loss \& disadvantage}}$ does not mediates the relationship between gaming addiction and hostility aggression. More gaming addiction predicts less drift rate in loss and risk-disadvantage condition, β = -0.018, *SE* = 0.004, $p < 0.001$, and a larger $v_{\text{loss \& disadvantage}}$ negatively predicts hostility aggression, β = -0.094, *SE* = 0.274, *p* = 0.730.

2.4 Self-aggression

For the mediation model, the direct effect of gaming addiction on self-aggression reach the significant level (β = 0.035, *SE* = 0.015, *p* = 0.017). The indirect path from gaming addiction to self-aggression through $v_{\text{loss \& disadvantage}}$ was not significant (indirect effect = -0.003, *p* = 0.353, 95% CI = [-0.013, 0.003]), indicating that the $v_{\text{loss \& disadvantage}}$ does not mediates the relationship between gaming addiction and self-aggression. More gaming addiction predicts less drift rate in loss and risk-disadvantage condition, β = -0.017, *SE* = 0.004, $p < .001$, and a larger $v_{\text{loss \& disadvantage}}$ negatively predicts verbal aggression, β = 0.020, *SE* = 0.209, *p* = 0.340.
